# Urinary ALCAM signatures define renal activity and therapeutic response in juvenile lupus nephritis

**DOI:** 10.1186/s12969-026-01193-9

**Published:** 2026-02-24

**Authors:** Dina Ebrahim Sallam, Mona ElGanzoury, Omar Osama Abdelnaby, Ahmed Mohamed Bakr, Sally Gouda Mohammed

**Affiliations:** 1 Department of Pediatrics and Pediatric Nephrology, Ain Shams University, Cairo, Egypt; 2 Department of Pediatrics and Pediatric Cardiology, Ain Shams University, Cairo, Egypt; 3 Department of Pediatrics, Ain Shams University, Cairo, Egypt; 4 Department of Histology and Cell Biology, Ain Shams University, Cairo, Egypt; 5 Department of Pediatrics and Pediatric Allergy, Immunology and Rheumatology, Ain Shams University, Cairo, Egypt

**Keywords:** Juvenile, Lupus nephritis, Biomarkers, Urinary ALCAM

## Abstract

**Background:**

Lupus nephritis (LN) remains a major cause of morbidity in childhood-onset systemic lupus erythematosus (SLE). Reliable non-invasive biomarkers for early diagnosis and disease monitoring are still limited. Activated leukocyte cell adhesion molecule (ALCAM) has emerged as a potential urinary biomarker reflecting renal immune injury. This study evaluated the diagnostic and monitoring performance of urinary ALCAM in juvenile LN and its correlation with disease activity scores, renal parameters, histopathological classes and indices.

**Methods:**

A controlled cross-sectional and prospective cohort study was conducted on 90 participants. Urinary ALCAM was measured in 60 pediatric patients with SLE, classified into 30 active LN and 30 active SLE non-LN patients, along with 30 age- and sex-matched healthy controls. Follow-up samples were obtained after 3 months of treatment in the active LN group. Associations with disease activity scores (SLEDAI and renal SLEDAI), renal function tests, histopathological classes, and renal pathology activity and chronicity indices were assessed. Diagnostic and monitoring performances of urinary ALCAM were analyzed using ROC curves.

**Results:**

Baseline urinary ALCAM levels were markedly higher in active LN (1001.6 [766.4–1186] ng/mg) compared with active SLE non-LN (229.7 [182–270.5] ng/mg) and controls (38.75 [25–54.5] ng/mg), with a *p* < 0.001. Levels declined significantly after treatment to 163.65 [85.6–238.2] ng/mg, with a *p* < 0.001. Urinary ALCAM correlated positively with serum creatinine, blood urea, proteinuria, SLEDAI, and renal SLEDAI scores, while negatively with estimated glomerular filtration rate. ROC analysis showed excellent diagnostic performance of urinary ALCAM. A cut-off of > 114.6 ng/mg (AUC = 0.994, sensitivity of 93.33% and specificity of 100%) differentiated juvenile SLE patients from healthy controls. A cut-off of > 538.7 ng/mg (AUC = 0.993, sensitivity and specificity of 96.67%) distinguished patients with LN from SLE non-LN. The optimal cut-off for differentiating active LN from remission was **≤** 407 ng/mg **(**AUC = 0.989, sensitivity of 86.67%, and specificity of 100%).

**Conclusion:**

Urinary ALCAM is a reliable, sensitive and non-invasive biomarker that accurately reflects renal activity and treatment response in juvenile LN. Its strong correlations with disease activity scores and renal functions, along with high diagnostic precision across clinically relevant cut-offs, underscore its potential as a practical tool for early detection, longitudinal monitoring of renal involvement, and guiding treatment decisions particularly when renal biopsy is not feasible and also, reducing reliance on serial biopsies.

## Introduction

Systemic lupus erythematosus (SLE) is a complex, autoimmune, multisystem disorder with a significant impact on the affected child or adolescent under 18 years of age. Despite sharing similar pathogenesis with adult onset SLE, the clinical presentation of SLE in pediatrics is generally more severe, with higher disease activity and damage, requiring early aggressive treatment [1]. Among its various clinical features, lupus nephritis (LN) is one of the most serious manifestations, occurring in approximately 50–75% of juvenile SLE patients and representing a great burden of morbidity and lower survival in these patients [2].

Renal involvement in juvenile SLE is often more aggressive than in adults, with a higher incidence of proliferative forms and a greater likelihood of progression to end-stage renal disease. Accurate and timely identification of LN activity is essential to optimize treatment and prevent irreversible damage. Renal biopsy remains the gold standard for adequate diagnosis, classification, assessment of activity and chronicity indices in LN and predicting prognosis [3]. However, its invasive nature, limited feasibility for serial monitoring, and potential risks especially during follow-up necessitate the search for reliable, non-invasive biomarkers that could accurately reflect renal pathology, track renal activity, guide treatment decisions and portend long-term prognosis [4].

Also, despite their widespread clinical use, conventional biomarkers in SLE have well-recognized limitations. Proteinuria may persist as a marker of structural renal damage rather than active inflammation, while anti-double-stranded DNA (anti-dsDNA) antibodies lack organ specificity and do not consistently correlate with renal activity. Collectively, these constraints highlight the need for novel biomarkers directly involved in immune cell–endothelial interactions, expressed at sites of active inflammation and could accurately reflect immunologically active renal disease [5].

Activated Leukocyte Cell Adhesion Molecule (ALCAM, also known as CD166) is a cell surface glycoprotein belonging to the immunoglobulin superfamily [6]. Soluble ALCAM in body fluids is produced through proteolytic cleavage by ADAM17 or alternative splicing [7].

It plays an essential role in leukocyte trafficking, T-cell activation, and endothelial adhesion, processes central to the pathogenesis of immune-mediated renal injury. Recent studies have suggested that ALCAM expression is upregulated in inflamed renal tissues and may be released into urine in response to renal tubular and glomerular injury [8]. The ability of urinary ALCAM to differentiate LN histopathology might be useful in the situation where renal biopsy is contraindicated or when patients are reluctant to do a biopsy. Such markers ideally should be capable of predicting early sub-clinical flares and could be used to follow response to therapy [9]. In adult SLE, elevated urinary ALCAM levels have been linked to histological activity and responsiveness to therapy; however, pediatric data remain scarce.

This study aimed to evaluate urinary ALCAM as a non-invasive biomarker for assessing renal disease activity and distinguishing active from remission states in juvenile LN. Furthermore, we aimed to determine its associations with disease activity scores, renal function, and histopathological features.

## Methods

### Study design and participants

This was as a controlled cross-sectional (Phase I) and prospective cohort (Phase II) study conducted at the Pediatric Allergy, Immunology and Rheumatology clinic, Children’s Hospital, Ain Shams University, Cairo, Egypt, during the period from May 2024 to January 2025. A total of 90 children and adolescents were enrolled and divided equally into three groups; 30 active SLE patients without renal involvement (active SLE non-LN group) with Systemic Lupus Erythematosus Disease Activity Index (SLEDAI-2 K) score ≥ 6 [10] and renal SLEDAI score = 0 [11], 30 active SLE patients with biopsy-proven LN and renal SLEDAI score ≥ 4 (active LN group) and 30 age- and sex-matched healthy controls (control group). Patients with a clinical diagnosis of SLE were enrolled if they fulfilled the 1997 revised American College of Rheumatology (ACR) [12] and/or the 2012 Systemic Lupus International Collaborating Clinics (SLICC) classification criteria of SLE [13], which were applied as eligibility criteria for inclusion in this study. Patients with autoimmune diseases rather than SLE, any chronic diseases (diabetes mellitus, hepatitis, tuberculosis) and patients with end-stage renal disease on regular hemodialysis were excluded from the study.

### Sample size calculation

A priori sample size calculation was conducted using Power Analysis and Sample Size (PASS) version 15 based on published data [14]. Assuming a moderate effect size (f = 0.40), a significance level of 0.05, and 90% power, a total sample size of 90 participants (30 per group) was required to detect differences in urinary ALCAM levels among three groups using one-way ANOVA. For the prospective cohort phase, at least 30 LN patients were included to evaluate the biomarker’s association with disease activity.

### Clinical and laboratory assessment

#### Initial evaluation (Phase I)

All enrolled patients underwent comprehensive clinical evaluation with detailed history taking including demographic data, age at onset and diagnosis, duration of SLE and diagnosis lag. Also, duration and presenting manifestations of LN if present (edema, hematuria, oliguria, and hypertension). Laboratory investigations comprised complete blood count (CBC), erythrocyte sedimentation rate (ESR), renal function (serum creatinine, blood urea and estimated glomerular filtration rate (eGFR) using the Schwartz formula). Immunological markers included anti-dsDNA antibodies, complement components (C3 and C4), and antiphospholipid antibodies (lupus anticoagulant, anticardiolipin and B2 glycoprotein IgG/IgM). Urinalysis was done for albumin, red and white cells and casts, also 24-hour urinary protein excretion was quantified. Global disease activity was assessed by the SLEDAI-2 K score [10] and renal activity by renal SLEDAI score [11]. Histopathological examination was performed in all active LN patients and classified using the 2004 International Society of Nephrology/Renal Pathological Society (ISN/RPS) classification [15], activity (0–24) and chronicity (0–12), indices of renal pathology were calculated.

#### Follow-up evaluation (Phase II)

Patients with active LN were reassessed 3 months after initiation or adjustment of immunosuppressive therapy. Renal symptoms, renal function, immunological markers, urine examination, and 24-hour urinary protein as well as global disease and renal activity scores were re-evaluated.

### Measurement of urinary ALCAM

Urinary ALCAM levels with Creatinine Parameter Assay Kit (KGE005, R&D Systems, Minneapolis, MN) were measured for both patient and control groups. For active LN patients, urine samples were obtained at the time of renal biopsy and compared with histopathological findings. A follow-up urine sample was collected after 3 months of intensified treatment in patients with active LN to assess changes in urinary ALCAM levels. All reagents and samples were equilibrated to room temperature prior to use. Mid-stream urine samples were collected in sterile containers. Samples were centrifuged at 3,000 rpm for 10 min to remove cellular debris and particulate matter. The supernatant was aliquoted and stored at − 80 °C until analysis. Repeated freeze–thaw cycles were avoided. No protease inhibitors were added, as samples were processed and frozen promptly after collection. Standards and samples were pipetted into the microplate wells pre-coated with specific capture antibodies, followed by sequential incubation with biotinylated anti-ALCAM antibody and streptavidin–HRP conjugate. After washing, colour development was achieved using substrate solutions A and B, and the reaction was terminated with stop solution. Optical density (OD) was measured at 450 nm using a microplate reader within 10 min of reaction termination. Urinary ALCAM levels were measured using a commercially available human enzyme-linked immunosorbent assay (ELISA) kit, which has been analytically validated by the manufacturer for quantitative determination in human urine samples. According to the manufacturer’s specifications, the intra-assay coefficient of variation (CV) for the ALCAM ELISA kit ranged from 3.2% to 3.5%, and the inter-assay CV ranged from 4.0% to 5.5%, indicating acceptable assay precision.

### Statistical analysis

Data were coded, tabulated, and analyzed using Statistical package for Social Science (SPSS 27). Quantitative variables were expressed as mean ± standard deviation (SD) or median **(**interquartile range, IQR) according to their distribution, while qualitative variables were presented as frequency and percentage. Comparisons between two independent groups were performed using the Student’s *t***-**test for parametric normally distributed data or the Mann–Whitney *U*-test for non-parametric not normally distributed data. Differences among more than two groups were assessed using one**-**way ANOVA or the Kruskal–Wallis test, followed by post hoc analyses when applicable. Associations between categorical variables were evaluated using the Chi-square test or Fisher’s exact test when expected frequencies were < 5. The paired *t***-**test was used to compare related quantitative variables. For paired categorical data, changes in binary variables were analyzed using the McNemar test, while the marginal homogeneity test was applied for multi-category variables measured at two time points. Repeated ordinal or non-parametric data measured at more than two time points were evaluated using the Friedman test. Correlations between continuous numerical variables were assessed using Spearman’s rank correlation coefficient. The receiver operating characteristic (ROC) curve analysis was performed to determine the optimal cut-off value and to evaluate the diagnostic and monitoring performance, including sensitivity and specificity, with the area under the curve (AUC) representing test accuracy. All statistical tests were two**-**tailed, and a *p*-value < 0.05 was considered statistically significant.

## Results

### Study population

Ninety participants were included; 30 patients with active SLE without nephritis (non-LN), 30 patients with biopsy-proven active LN (active LN), and 30 age-and sex-matched healthy controls. The mean age was comparable across the three groups (*p* = 0.069). Females predominated in both patient groups (83.3% in non-LN, 80% in LN) compared to controls (63.3%). Systolic and diastolic blood pressure Z-scores were markedly higher in active LN patients (median SBP 92.5 [85–98]; DBP 94 [81–97]) compared with active SLE non-LN and control groups (*p* < 0.001).

#### 1. Comparison between active SLE with and without LN

##### 1.a. Demographic data and disease characteristics

Active LN patients had a later disease onset (11.76 ± 2.27 vs. 9.65 ± 3.07 years, *p* = 0.004), later diagnosis (12.29 ± 1.98 vs. 10.11 ± 3.06 years, *p* = 0.002) and shorter disease duration (6 [3–16] vs. 21 [5–36] months, *p* = 0.025) compared to non-LN patients. Although multisystem involvement (haematological, pulmonary, cardiac, neurological, and musculoskeletal) was comparable; 36.7% in active SLE non-LN and 26.67% in active LN patients, however, the global disease activity (SLEDAI) was significantly higher in the active LN group (12 [10–14]) compared to non-LN patients (9 [8–12], *p* < 0.001) (Table 1).

##### 1.b. Routine and immunological laboratory investigations

Routine laboratory investigations including blood counts and ESR did not differ significantly between both groups. However, active LN patients had notably worse renal function, with higher serum creatinine (0.66 ± 0.21 vs. 0.46 ± 0.15 mg/dL, *p* < 0.001) and elevated urea levels (43 vs. 19.5 mg/dL, *p* < 0.001) along with lower eGFR (116.5 vs. 162.55 ml/min/ 1.73m^2^, *p* = 0.001) compared to active SLE non-LN patients. Additionally, the 24-hour urinary protein excretion was markedly elevated in the active LN group compared to the non-LN group (610 vs. 120 mg/day, *p* < 0.001). Regarding the immunological markers, anti-dsDNA was positive in 24 patients (80%) of both groups. C3 levels were lower in active LN patients (42 [30.1–55] vs. 52.5 [36–74.2] mg/L), while the anticardiolipin IgG levels were significantly higher in active SLE non-LN patients (6.9 [2.3–31.2] vs. 3.2 [1.5–10] U/mL, *p* = 0.033) (Table 1).

##### 1.c. Renal histopathology of active LN group

Renal biopsy findings in the active LN group revealed diverse histopathological patterns, with mesangial proliferative LN being the most common, found in 12 patients (40%), followed by focal LN either proliferative or sclerosing or both in 6 patients (20%). While diffuse proliferative and sclerosing LN and minimal mesangial LN were found in 5 patients each (16.67%). Only two patients (6.67%) had membranous LN. The chronicity index had a median (IQR) of 2 (1–3), while the activity index had a median (IQR) of 5.5 (2–9).

##### 1.d. Therapeutic profiles

The immunosuppressive medications used showed key differences between active SLE non-LN and active LN groups. Corticosteroids were the mainstay therapy used by all patients in both groups. Cyclophosphamide was used more frequently by the non-LN group (33.33% vs. 3.33%, *p* = 0.006), whereas mycophenolate mofetil (MMF) was significantly more common in the active LN group (73.33% vs. 36.67%, *p* = 0.004). Other immunosuppressive drugs including azathioprine, calcineurin inhibitors and rituximab showed no significant differences between the two groups.

**Table 1:** Disease characteristics, laboratory investigations and urinary ALCAM between active SLE non-LN and active LN groups

Patients Groups			Active SLE non-LN (*N* = 30)		Active LN (*N* = 30)	*p*-value
**Age at onset (years)** Mean ± SD			9.65 ± 3.07		11.76 ± 2.27	**0.004**
**Age at diagnosis (years)** Mean ± SD			10.11 ± 3.06		12.29 ± 1.98	**0.002**
**Gender** N (%)		**Male**	5 (16.67%)		6 (20%)	0.739
		**Female**	25 (83.33%)		24 (80%)	
**Diagnosis lag (months)** Median (IQR)			5.5 (2–12)		2 (1–4)	0.110
**Duration of SLE (months)** Median (IQR)			21 (5–36)		6 (3–16)	**0.025**
**Organ involvement rather than LN**		**No**	19 (63.3%)		22 (73.33%)	0.405
		**Yes**	11 (36.7%)		8 (26.67%)	
**Type of Organ involvement** N (%)		**Hematological**	2 (18.2%)		0 (0%)	0.485
		**Pulmonary**	1 (9.1%)		0 (0%)	1.00
		**Cardiac**	6 (54.5%)		4 (50%)	1.00
		**CNS**	2 (18.2%)		2 (25%)	1.00
		**MSK**	1 (9.1%)		2 (25%)	0.546
**SLEDAI Score** (10 days before enrolment) Median (IQR)			9 (8–12)		12 (10–14)	**< 0.001**
**Routine laboratory investigations**						
**TLC x 10**^**3**^ **/µL** Median (IQR)			7.35 (4.3–9.8)		6.85 (5.3–8.5)	0.779
**ANC x 10**^**3**^ **/µL** Median (IQR)			3.5 (2.4–7.2)		3.3 (2.9–5.6)	0.717
**ALC x 10**^**3**^ **/µL** Median (IQR)			1.68 (0.8–2.1)		2 (1.2–2.3)	0.176
**Hgb (g/dL)** Mean ± SD			10.51 ± 2.42		10.47 ± 1.68	0.941
**PLT x 10**^**3**^ **/µL** Median (IQR)			218.5 (116–338)		224 (95–271)	0.277
**ESR (mm/hr)** Median (IQR)			67.5 (50–80)		72.5 (53–90)	0.424
**S. creatinine (mg/dl)** Mean ± SD			0.46 ± 0.15		0.66 ± 0.21	**< 0.001**
**Urea (mg/dl)** Median (IQR)			19.5 (16–30)		43 (28–58)	**< 0.001**
**GFR (Schwartz formula) (ml/min/1.73 m** ^**2**^ **)** Median (IQR)			162.5 (130–210)		116.5 (99.6–137)	**0.001**
**24 h Urinary proteins (mg/day)** Median (IQR)			120 (66–146)		610 (390–830)	**< 0.001**
**Immunological markers**						
**Anti ds DNA (IU/mL)** N (%)	**Negative**			6 (20%)	5 (16.67%)	1.00
	**Borderline**			0 (0%)	1 (3.33%)	
	**Positive**			24 (80%)	24 (80%)	
**C3 (mg/L)** Median (IQR)				52.5 (36–74.2)	42 (30.1–55)	0.201
**Lupus anticoagulant** Median (IQR)				29.75 (21–35)	32.75 (22–40.2)	0.506
**Anticardiolipin (IgM)** **(U/ml)** Median (IQR)				7.15 (1.5–16)	5.1 (2.1–10)	0.953
**Anticardiolipin (IgG)** **(U/ml)** Median (IQR)				6.9 (2.3–31.2)	3.2 (1.5–10)	**0.033**
**Anti ß2 glycoprotein (IgM)** **(U/mL)** Median (IQR)				1.8 (1–6)	2.95 (1.9–6)	0.126
**Anti ß2 glycoprotein (IgG)** **(U/mL)** Median (IQR)				9.1 (1.6–33)	5.5 (1.2–12)	0.122
**Urinary ALCAM**						
**Urinary ALCAM** **(ng/mg)** Median (IQR)				229.7 (182-270.5)	1001.6 (766.4–1186)	**< 0.001**

LN : lupus nephritis, SLE: systemic lupus erythematosus, CNS: central nervous system, MSK: musculoskeletal system, SLEDAI: Systemic Lupus Erythematosus Disease Activity Index, TLC: total leucocytic count, ANC: absolute neutrophil count, ALC: absolute lymphocytic count, Hgb: haemoglobin, PLT: platelets, ESR: erythrocyte sedimentation rate, GFR: glomerular filtration rate, Anti-ds DNA: anti-double stranded DNA antibodies, C3: complement 3, ALCAM : activated leukocyte cell adhesion molecule, SD: standard deviation, IQR: interquartile range

### Urinary ALCAM levels across study groups

**Fig. 1:**
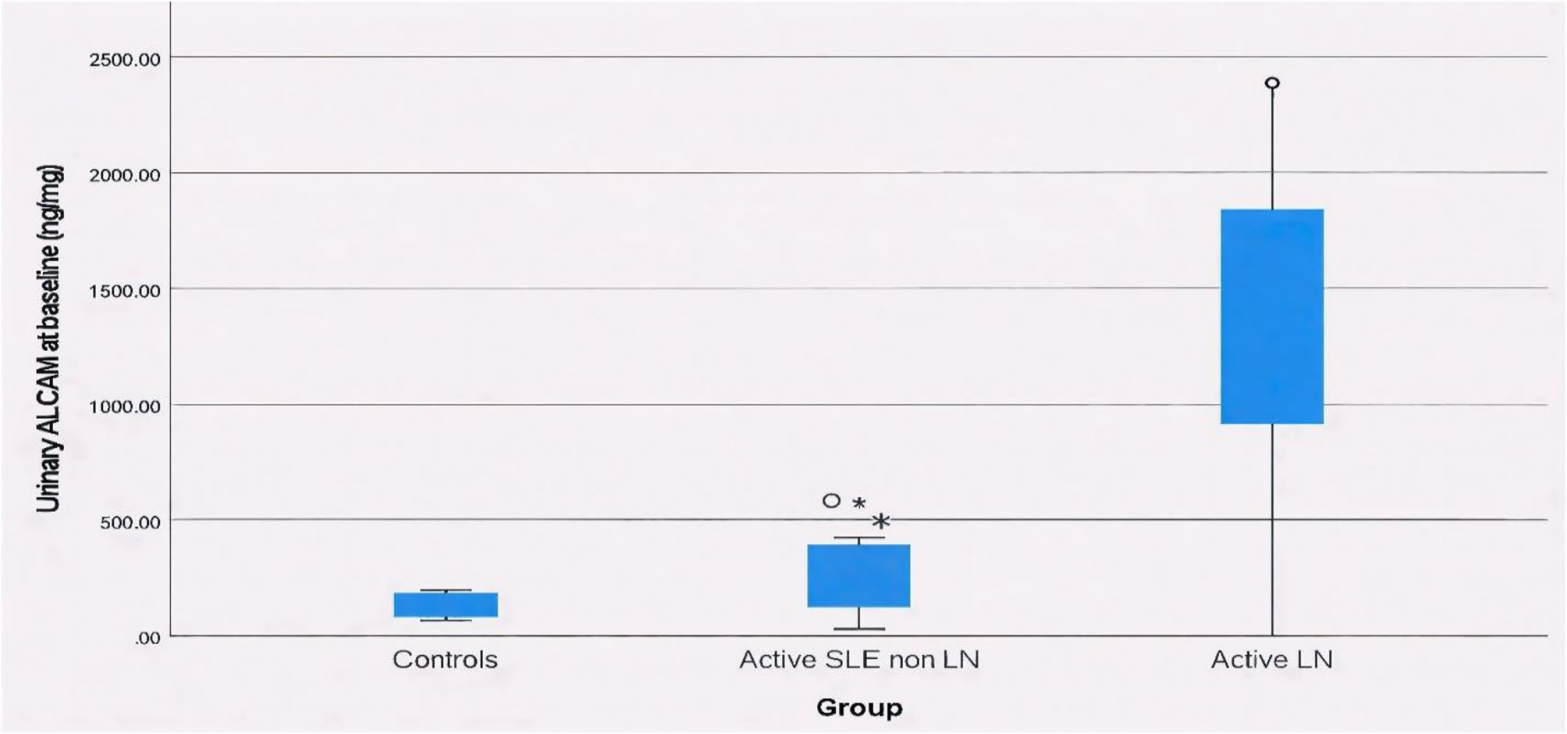
Urinary ALCAM among the three groups

**Fig. 2:**
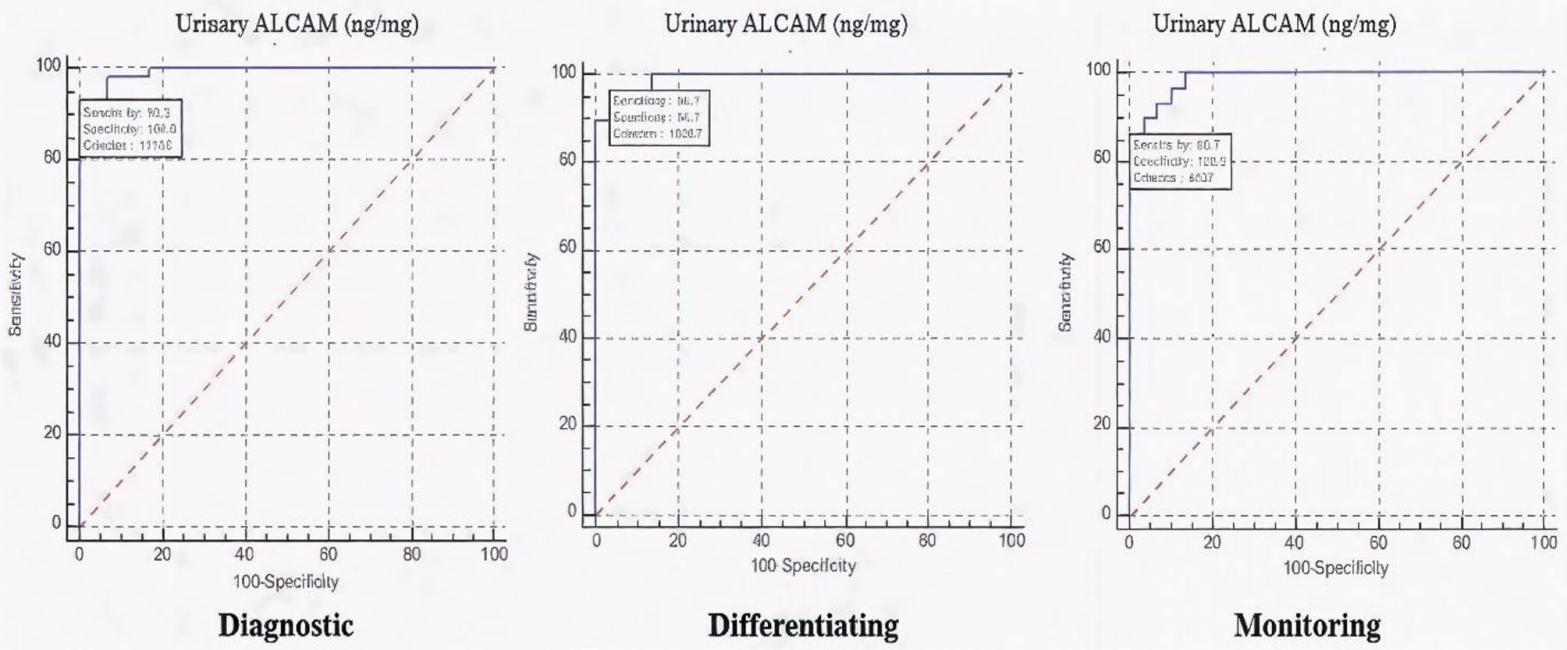
ROC curve showing different urinary ALCAM roles as a diagnostic, differentiating and monitoring biomarker

Urinary ALCAM levels showed a striking increase across the three studied groups, with a median (IQR) of 38.75 (25–54.5) ng/mg in controls, significantly rising to 229.7 (182–270.5) ng/mg in active SLE non-LN, and 1001.6 (766.4–1186) ng/mg in active LN groups with a *p* < 0.001 (Fig. 1). Receiver operating characteristic (ROC) curve analysis revealed that urinary ALCAM at a cut-off value of > 114.6 ng/mg, was an excellent discriminator between SLE patients and healthy controls, with an AUC of 0.994, sensitivity of 93.33% and specificity of 100%. While at a cut-off value of > 538.7 ng/mg, urinary ALCAM was also an excellent marker differentiating active SLE non-LN from active LN patients, with AUC of 0.993, sensitivity and specificity of 96.67%, and both positive and negative predictive values of 96.7% (Fig. 2).

#### 2. Follow-up of active LN patients (at 3-month reassessment)

##### 2.a. Renal features and disease activity at baseline and at follow-up

At 3-month follow-up of active LN patients, the renal symptoms showed significant improvement. Edema present in 90% of patients at baseline, dramatically decreased to 3.33% with a *p* < 0.001, while oliguria resolved completely (23.33% to 0%), with a *p* = 0.016. Hypertension also showed a significant reduction from 73.33% to 40% with a *p* = 0.006, however, gross hematuria was absent both at baseline and at follow-up. The global disease activity also showed marked improvement, with median (IQR) SLEDAI scores declining from 12 (10–14) to 3 (0–4) with a *p* < 0.001, while median (IQR) renal SLEDAI scores decreased from 8 (4–12) to 0 (0–4) with a *p* < 0.001, corresponding to median reductions of 81.67% and 100%, respectively (Table 2).

##### 2.b. Routine and immunological investigations at baseline and at follow-up

Hematologic recovery was evident with significant rises in hemoglobin levels (10.47 ± 1.68 to 11.31 ± 1.32 g/dL) and platelet counts (95–271 × 10^3^/µL) to (255–319 × 10^3^/µL) with *p* = 0.034 and 0.007, respectively. Moreover, the median ESR markedly decreased (72.5 to 16.5 mm/hr). Regarding renal function, there was a significant decrease in both serum creatinine (0.66 ± 0.21 to 0.52 ± 0.12 mg/dl) and blood urea (43 to 23 mg/dl) with *p* = 0.001 and < 0.001, respectively, along with an increase in eGFR (116.5 to 163.1 ml/min/1.73m^2^). Additionally, proteinuria significantly improved, and decreased (610 to 150 mg/day). The immunological markers also showed marked improvement at follow-up. Positive anti-dsDNA results decreased from 80% to 20%, with a corresponding rise in negative results from 16.67% to 80% with a *p* < 0.001. Additionally, C3 levels significantly increased from a median of 42 to 106.95 mg/L with a *p* < 0.001) (Table 2).

##### 2.c. Urine examination and urinary ALCAM at baseline and at follow-up

**Fig. 3:**
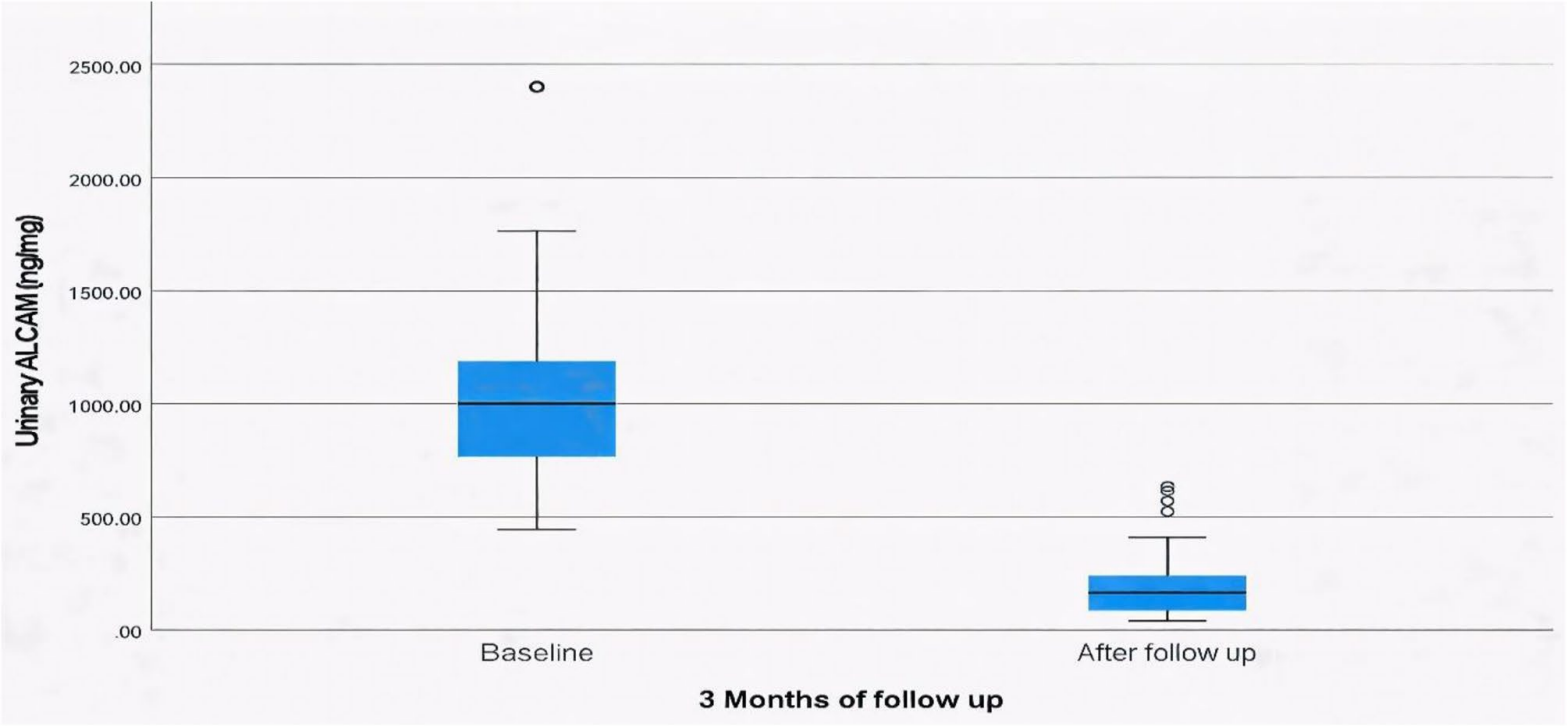
Urinary ALCAM in active LN patients at baseline and at follow up

A notable improvement was observed in the urinary tests of active LN patients at follow-up. Pyuria decreased from 40% to 13.33% with a *p* = 0.039, and albuminuria, present in 53.33% at baseline, completely resolved in all patients with a *p* < 0.001. Additionally, urinary casts, initially observed in 30% of cases, disappeared entirely after treatment with a *p* = 0.004. While microscopic hematuria showed a reduction from 23.33% to 10%, but no significant difference was found. Urinary ALCAM levels in the active LN group showed a marked decline following treatment. Median (IQR) urinary ALCAM levels decreased from 1001.6 (766.4–1186) ng/mg at baseline to 163.65 (85.6–238.2) ng/mg at follow-up (p **<** 0.001) (Fig. 3). At a cut-off of **≤** 407 ng/mg, urinary ALCAM was an excellent marker for differentiating remission from active disease with an AUC of 0.989, sensitivity of 86.67% and specificity of 100% (Fig. 2).

**Table 2:** Renal features, activity scores, laboratory investigations and urinary ALCAM in active LN patients at baseline and at follow-up

LN group (*N* = 30)			Baseline (*N* = 30)	Follow up (*N* = 30)	*p*-value
**Renal symptoms** N (%)					
**Edema** **Oliguria** **HTN** **Gross hematuria**			27 (90%)	1 (3.33%)	**< 0.001**
			7 (23.33%)	0 (0%)	**0.016**
			22 (73.33%)	12 (40%)	**0.006**
			0 (0%)	0 (0%)	
**Routine laboratory investigations**					
**TLC x 10**^**3**^ **/µL** Median (IQR)			6.85 (5.3–8.5)	7.25 (4.9–9.2)	0.837
**ANC x 10**^**3**^ **/µL** Median (IQR)			3.3 (2.9–5.6)	4.15 (2.7–6.7)	0.726
**ALC x 10**^**3**^ **/µL** Median (IQR)			2 (1.2–2.3)	1.85 (1.3–2.4)	0.681
**Hgb (g/dL)** Mean ± SD			10.47 ± 1.68	11.31 ± 1.32	**0.034**
**PLT x 10**^**3**^ **/µL** Median (IQR)			224 (95–271)	285 (255–319)	**0.007**
**ESR (mm/hr)** Median (IQR)			72.5 (53–90)	16.5 (12–20)	**< 0.001**
**S. creatinine (mg/dl)** Mean ± SD			0.66 ± 0.21	0.52 ± 0.12	**0.001**
**Urea (mg/dl)** Median (IQR)			43 (28–58)	23 (19–30)	**< 0.001**
**GFR (Schwartz formula) (ml/min/1.73m2)** Median (IQR)			116.5 (99.6–137)	163.1 (125–180)	**0.001**
**24 h Urinary proteins (mg/day)** Median (IQR)			610 (390–830)	150 (110–260)	**< 0.001**
**Immunological markers**					
**Anti ds DNA (IU/mL)** N (%)	**Negative**		5 (16.67%)	24 (80%)	**< 0.001**
	**Borderline**		1 (3.33%)	0 (0%)	
	**Positive**		24 (80%)	6 (20%)	
**C3 (mg/L)** Median (IQR)			42 (30.1–55)	106.95 (95.7–137)	**< 0.001**
**Urine examination**					
**Pyuria** N (%)		**No**	18 (60%)	26 (86.67%)	**0.039**
		**Yes**	12 (40%)	4 (13.33%)	
**Microscopic hematuria** N (%)		**No**	23 (76.67%)	27 (90%)	0.289
		**Yes**	7 (23.33%)	3 (10%)	
**Albuminuria** N (%)		**Nil**	14 (46.67%)	30 (100%)	**< 0.001**
		**(+)**	11 (36.67%)	0 (0%)	
		**(++)**	3 (10%)	0 (0%)	
		**(++++)**	2 (6.67%)	0 (0%)	
**Casts** N (%)		**No**	21 (70%)	30 (100%)	**0.004**
		**Granular**	9 (30%)	0 (0%)	
**Activity scores**					
**SLEDAI Score** (10 days before enrolment) Median (IQR)			12 (10–14)	3 (0–4)	**< 0.001**
**Renal SLEDAI Score** Median (IQR)			8 (4–12)	0 (0–4)	**< 0.001**
**Urinary ALCAM**					
**Urinary ALCAM** **(ng/mg)** Median (IQR)			1001.6 (766.4–1186)	163.65 (85.6–238.2)	**< 0.001**

LN: lupus nephritis, HTN: hypertension, SLEDAI: Systemic Lupus Erythematosus Disease Activity Index, TLC: total leucocytic count, ANC: absolute neutrophil count, ALC: absolute lymphocytic count, Hgb: hemoglobin, PLT: platelets, ESR: erythrocyte sedimentation rate, GFR: glomerular filtration rate, Anti-ds DNA: anti-double stranded DNA antibodies, C3: complement 3, ALCAM: activated leukocyte cell adhesion molecule, SD: standard deviation, IQR: interquartile range.

### Correlations between urinary ALCAM and renal parameters and activity scores

**Fig. 4:**
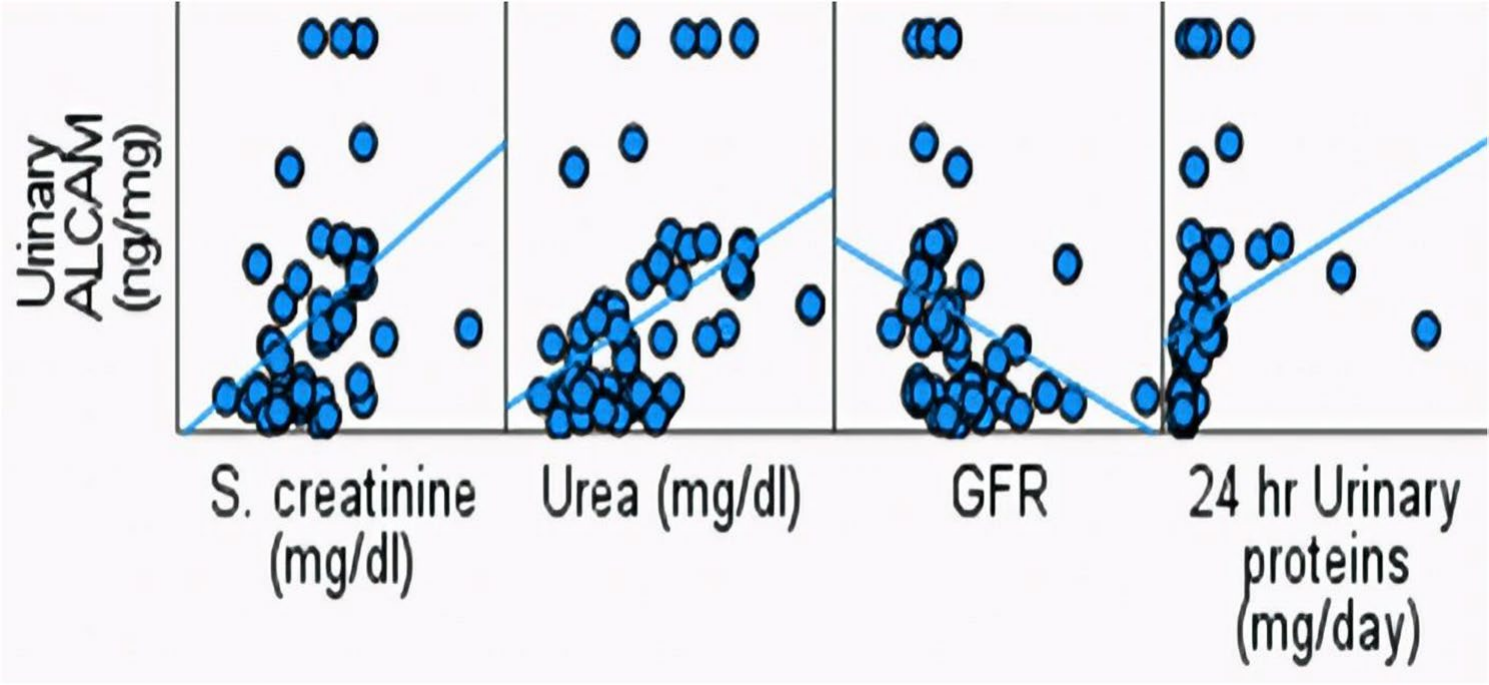
Correlation between urinary ALCAM and renal function parameters and proteinuria

**Fig. 5:**
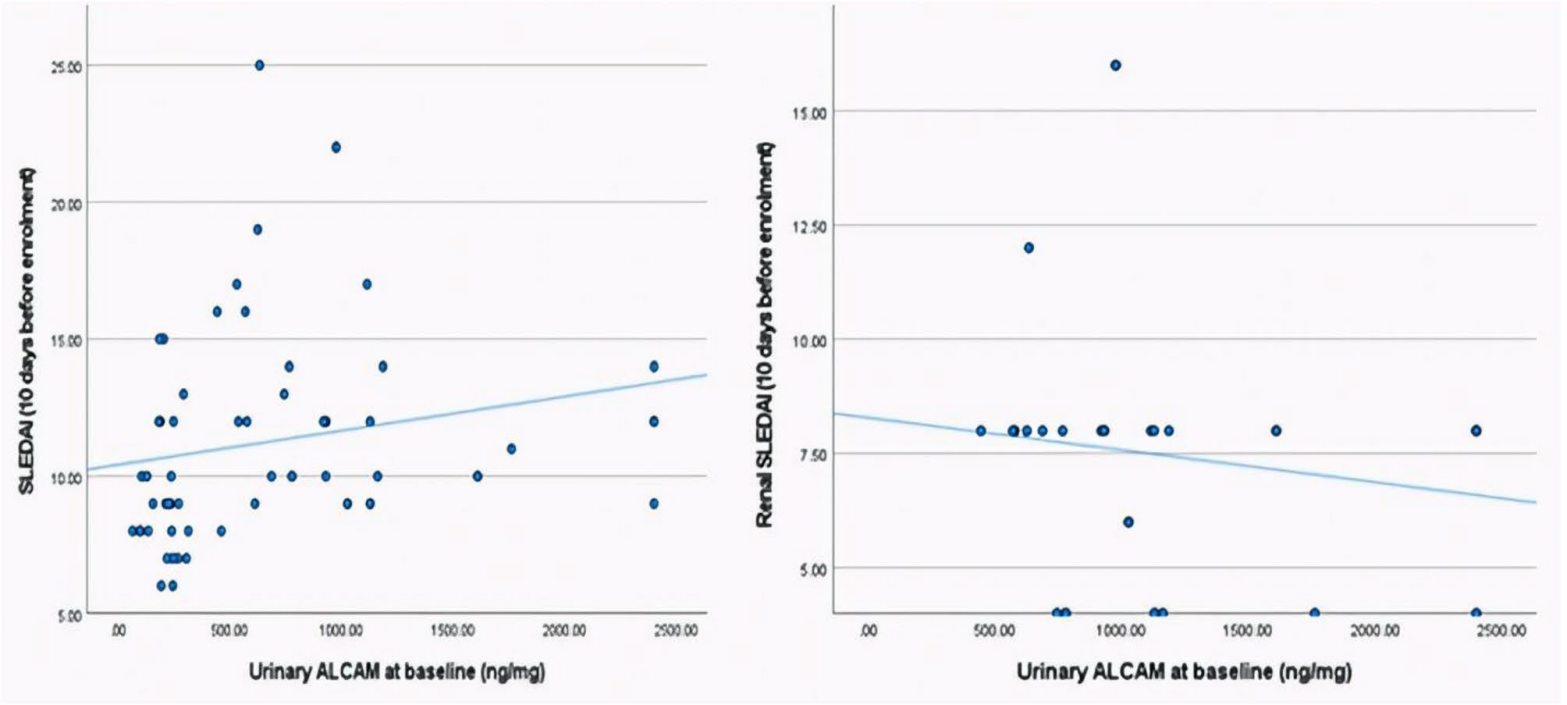
Correlation between urinary ALCAM and activity scores

In patients with active LN, urinary ALCAM at baseline showed strong positive correlations with renal function parameters, including serum creatinine, blood urea and 24-hour urinary protein excretion (*p* < 0.001), and a negative correlation with eGFR (*p* = 0.003) (Fig. 4). Additionally, urinary ALCAM correlated significantly with global SLE disease activity (SLEDAI) and renal disease activity (renal SLEDAI), with *p* = 0.005 and < 0.001, respectively (Fig. 5).

### Association of urinary ALCAM with histopathological classes and indices

When urinary ALCAM levels were analyzed across histopathological classes of LN, patients with proliferative LN (classes III and IV) showed higher urinary ALCAM levels (1027 [976.2–1762] ng/mg), compared to those with mesangial LN (classes I and II) who had urinary ALCAM values (919.3 [743.7–1186] ng/mg), or membranous LN (class V) (655.9 [625–686.8] ng/mg), though this did not reach a significant difference (*p* > 0.07). Additionally, no correlation was found between urinary ALCAM and renal biopsy activity or chronicity indices.

## Discussion

Lupus nephritis (LN) continues to represent one of the most serious and prognostically significant manifestations of SLE, particularly in children and adolescents, where it carries significant morbidity and long-term renal risk. Though kidney biopsy remains the gold standard for diagnosing and assessing LN, however, it is an invasive procedure associated with potential risks, therefore, identifying non-invasive biomarkers that can mirror renal activity and remission more precisely is essential [14]. Although significant efforts have been directed toward identifying reliable biomarkers for the diagnosis and prognosis of LN, a definitive solution has yet to be achieved. The complex and heterogeneous nature of the disease continues to hinder their routine clinical application. Therefore, there remains a pressing need for more accurate and specific biomarkers to improve diagnostic precision and predict therapeutic outcomes in LN [16]. Among the emerging candidates, ALCAM has emerged as a promising biomarker for assessing renal activity, where its role in reflecting renal inflammation and disease progression has gained attention due to its established role in leukocyte trafficking, cellular adhesion and immune regulation [14].

Urinary ALCAM plays a pivotal role in inflammatory processes by contributing to T-cell co-stimulation and facilitating the recruitment of activated monocytes and T cells. When renal injury occurs, ALCAM promotes the release of inflammatory cytokines, which further attract immune cells such as T cells, monocytes, inflammatory dendritic cells, neutrophils, and B cells [17, 18]. During lupus-related inflammation, this pathway enhances cytokine production and results in increased urinary excretion of ALCAM, a feature especially evident in patients with LN [19].

In the present study, urinary ALCAM was evaluated in juvenile SLE patients to explore its diagnostic and disease-monitoring value in juvenile SLE. Our findings revealed markedly higher urinary ALCAM levels in children with active LN compared with those with both active SLE non-LN and healthy controls. This significant elevation in urinary ALCAM among active LN patients reflects its close link to renal inflammatory activity rather than systemic disease activity alone. The observed increase likely reflects enhanced local production and shedding from activated tubular and endothelial cells within inflamed glomeruli. These findings are in agreement with those of Ding et al. and Soliman et al. who reported significantly higher urinary ALCAM levels in active LN cases compared to non-renal SLE cases and healthy controls [14, 20]. Moreover, in our study significant differences were observed in the age at onset and diagnosis, disease duration, and immunosuppressive therapy between both active LN and active SLE non-LN groups, which likely reflect underlying differences in disease phenotype but also represent potential confounders. Accordingly, we cannot exclude that these factors may have influenced urinary ALCAM levels. Nevertheless, the observed elevation of urinary ALCAM in LN was directionally consistent with its proposed role in renal immune activation rather than demographic or treatment-related effects alone. These findings should therefore be interpreted cautiously, and future studies using matched cohorts and longitudinal designs are warranted to clarify the independent contribution of renal inflammation to urinary ALCAM elevation.

In our study, the ROC curve analysis revealed optimal cut-off values with excellent sensitivity and specificity for various clinical scenarios. A threshold of urinary ALCAM > 114.6 ng/mg (AUC 0.994, *p* < 0.001) effectively distinguished juvenile SLE patients from healthy controls with 93.33% sensitivity and 100% specificity confirming its strong diagnostic potential for SLE in children. While, at a higher cut-off value > 538.7 ng/mg (AUC 0.993, *p* < 0.001), urinary ALCAM differentiated patients with LN from SLE non-LN with 96.67% sensitivity and specificity, reflecting also its ability to accurately identify renal involvement among juvenile lupus patients. The diagnostic performance of urinary ALCAM was exceptional, with AUC values exceeding 0.99. This may reflect rigorous phenotyping of the study population and the strong pathophysiological association of ALCAM with renal inflammation and immune activation in SLE. Nevertheless, such remarkably high AUC values warrant cautious interpretation, as they may partially reflect cohort-specific characteristics and controlled study conditions. Therefore, external validation in larger, independent, and multicenter cohorts is essential to confirm the robustness, generalizability, and clinical applicability of these findings. In the study by Amer et al., on 60 adult patients with SLE, urinary ALCAM differentiated SLE patients from healthy controls at a cut-off value of > 112.5 ng/mg, with a sensitivity of 86.7% and specificity of 85% (p *<* 0.001) [21]. While when comparing SLE patients with and without LN, a higher cut-off of > 196.5 ng/mg provided a sensitivity of 60% and specificity of 53.3%, (*p* = 0.012). Additionally, Soliman et al. in his study on 84 children (59 with juvenile SLE and 25 healthy controls) found that urine ALCAM showed good performance (AUC 0.75, *p* = 0.001) in discriminating renal disease activity among SLE patients (with sensitivity and specificity values ranging from 78 to 92%) [20].

Moreover, in our study there was a significant decline in urinary ALCAM levels following treatment in active LN patients. Median levels decreased from 1001.6 ng/mg at baseline to 163.65 ng/mg at follow-up (*p* < 0.001), paralleling clinical and laboratory improvement. This reduction signifies ALCAM’s responsiveness to therapeutic control and its potential as a dynamic biomarker reflecting changes in renal inflammation over time. The ROC analysis further substantiated its clinical utility, showing excellent discrimination between active disease and remission at a cut-off value of ≤ 407 ng/mg, with high sensitivity of 86.67%, and specificity of 100%. Such discriminative capability indicates that urinary ALCAM could complement conventional urinary and serological markers in assessing treatment response and determining remission, particularly in pediatric settings, reinforcing its clinical promise for non-invasive disease assessment.

Interestingly, our study revealed strong positive correlations between urinary ALCAM levels and key indicators of renal function, including serum creatinine, blood urea, and 24-hour urinary protein excretion, suggesting its potential association with renal dysfunction and increased proteinuria. Conversely, a significant negative correlation was found with eGFR, further supporting its potential role as a biomarker of declining kidney function. These relationships emphasize that urinary ALCAM reflects renal impairment severity. These findings are in agreement with Ding et al. who observed in a study on 256 adult Chinese with lupus a positive correlation between urinary ALCAM and 24-hour urine protein suggesting that ALCAM could be an indicator of renal damage in LN, however, no significant correlation was observed between urinary ALCAM and serum creatinine, indicating that the change in urinary ALCAM was not likely to be impacted by renal function [14].

In terms of disease activity, urinary ALCAM demonstrated a positive correlation with global and renal disease activity scores (SLEDAI and renal SLEDAI), highlighting the potential utility of urinary ALCAM as a non-invasive biomarker for assessing disease activity in juvenile LN patients. Ding et al. and Soliman et al. similarly reported strong positive correlations between urinary ALCAM and global and renal disease activity indices [14, 20].

In this study, when urinary ALCAM levels were analyzed in relation to histopathological classes of LN, higher values were detected in proliferative forms (classes III and IV), compared to mesangial (classes I and II) and membranous (class V) classes, although interclass differences did not reach significance, most probably due to the limited number of patients within each histologic category. Nevertheless, urinary ALCAM levels did not show a significant association with conventional histopathologic activity and chronicity indices of renal pathology. Our finding of discordance between urinary ALCAM and conventional histopathologic activity/chronicity indices aligns with emerging literature on LN biomarkers. Traditional indices are largely weighted toward glomerular lesions and overt structural changes, whereas emerging evidence suggests that tubulointerstitial inflammation and immune cell trafficking play a critical and sometimes independent role in LN pathogenesis and prognosis [22, 23]. Given that ALCAM is involved in leukocyte adhesion, transmigration, and T-cell activation, elevated urinary levels may preferentially reflect active tubulointerstitial immune signaling or endothelial–immune interactions rather than glomerular injury alone [8]. These data support the hypothesis that urinary biomarkers such as urinary ALCAM may be sensitive to dynamic inflammatory processes occurring at a molecular or cellular level and may detect early or diffuse immune activation before they become morphologically evident on light microscopy, while renal biopsy provides a static morphological assessment. Nevertheless, these findings do not diminish the central role of renal biopsy in LN but rather highlight its inherent limitations in capturing ongoing immunologic activity. Soliman et al. in his study found that urine ALCAM levels were not associated with proliferative LN or with renal pathology activity or chronicity indices, suggesting that the molecular determinants of clinical disease activity and renal disease activity in juvenile SLE may be distinct [20]. However, Ding et al. observed that urinary ALCAM (AUC = 0.81, *p* < 0.001) was significantly increased in patients with proliferative LN (14.10 IQR (18.21) ng/mg) compared to those with membranous LN (4.70 IQR (4.56) ng/mg) and that urinary ALCAM levels correlated positively with activity index (*r* = 0.405, *p* < 0.001) but not chronicity index (*r* = 0.08, *p* = 0.448) in renal histopathology [14].

Together, our findings position urinary ALCAM as a highly promising biomarker for its diagnostic, differentiating, and monitoring roles in juvenile LN. It not only differentiated LN from SLE non-LN but also discriminated active from quiescent disease states and was able to mirror renal recovery. Thus, urinary ALCAM could provide complementary information to support clinical evaluation, treatment planning and more precise monitoring of therapeutic outcomes.

Our study entails many strong points, where ALCAM was measured using ELISA, a well-established and standardized laboratory technique, ensuring the reliability and reproducibility of results. Also, the cross-sectional study design in phase I, enabled robust comparisons among different study groups, enhancing the validity of the findings and the prospective cohort study in phase II, identified the notable role of urinary ALCAM in detecting remission and being used as a biomarker not only for diagnosis but for predicting renal outcomes as well.

In conclusion, our findings demonstrated markedly elevated urinary ALCAM levels in active LN cases compared to non-LN and control groups, with significant reductions observed following treatment. In a child with SLE and new-onset proteinuria, a urinary ALCAM > 538.7 ng/mg could strongly support the decision to proceed with biopsy or initiate empiric therapy for LN if biopsy is delayed. During treatment, a drop to < 407 ng/mg could support the assessment of clinical remission. The diagnostic accuracy of urinary ALCAM, as shown by high sensitivity and specificity at various cut-off levels, underscores its utility in differentiating LN from other SLE states and monitoring treatment response. Furthermore, urinary ALCAM correlated strongly with disease activity scores and key indicators of renal dysfunction, including serum creatinine, blood urea, GFR, and proteinuria. Our study is limited by the single-center design and relatively homogenous population, limiting generalizability and the short follow-up period (3 months); multi-center with larger cohorts, longer follow-up is needed to assess urinary ALCAM’s utility in predicting long-term renal outcomes and relapse. Potential confounding by differing treatments between groups, as noted in results and the cross-sectional nature of the histopathology comparison; urinary ALCAM was measured at a single time point corresponding to biopsy.

## Data Availability

No datasets were generated or analysed during the current study.
